# Supplementary material for: Effect of Ultrasound and High Hydrostatic Pressure Processing on Quality and Bioactive Compounds during the Shelf Life of a Broccoli and Carrot By-Products Beverage
Source: Foods. 2023 Oct 17;12(20):3808. doi: 10.3390/foods12203808 (PMC10606312; doi:10.3390/foods12203808)
Supplement: Supplementary file 1 [file foods-12-03808-s001.zip › foods-2634567-supplementary.pdf]

Table S1. L*, a*, b*, ΔE, and pH changes during 28 days at 4 °C in a broccoli and carrot by-products beverage according to several processing treaments.

Processing Treatment	Parameter	0 d	3 d	8 d	15 d	22 d	28 d
CTRL	L*	45.7 a C	42.4 ab C	40.0 b B	44.0 b A	40.5 b	40.0 b
	a*	3.0 b B	3.5 ab	3.8 ab C	4.3 a AB	3.5 ab B	3.4 ab
	b*	19.0 a B	17.0 ab	15.1 ab B	16.4 ab B	15.9 b	15.8 b
	ΔE	0.0 d D	3.8 c B	6.2 a B	5.4 ab B	6.1 a AB	6.5 a B
	pH	6.2 a A	6.1 a	5.7 ab AB	5.4 ab B	5.0 b B	4.5 b B
P_90	L*	39.0 D	40.0 C	39.0 A	41.4 B	40.0	39.0
	a*	2.5 b B	3.3 a	4.4 a A	3.5 a CD	3.5 a B	3.5 a
	b*	16.9 C	16.9	17.1 AB	16.3 B	16.0	15.8
	ΔE	7.0 A	6.0 A	7.1 A	6.3 A	6.4 A	7.4 A
	pH	6.0 C	5.9	6.0 BC	5.8 A	5.9 A	5.5 A
HHP_500_10	L*	44.9 a BC	43.0 a BC	40.7 b AB	41.6 ab B	41.0 b	40.1 b
	a*	3.3 B	3.6	3.8 C	3.9 BCD	4.0 A	3.7
	b*	18.4 a B	17.6 a	16.4 ab AB	16.7 b AB	16.3 b	16.2 b
	ΔE	1.0 d C	3.1 c C	5.4 ab C	4.7 b C	5.5 a B	6.3 a B
	pH	6.0 BC	6.0	6.1 C	5.8 A	5.8 A	5.6 A
US_50_10	L*	45.1 a ABC	46.4 a ABC	40.8 b AB	41.8 b B	41.6 b	41.7 b
	a*	2.7 b C	3.4 a	4.0 a BC	3.6 a D	3.5 a B	3.5 a
	b*	18.0 a B	17.0 ab	16.5 ab AB	16.6 ab AB	16.5 ab	16.4 b
	ΔE	1.2 c C	2.2 b D	5.5 a C	4.6 ab C	4.8 ab C	4.8 ab C
	pH	6.2 a B	5.9 a	6.2 ab A	5.5 ab AB	5.1 b B	4.7 b B
US_50_5+ HHP_500_5	L*	46.4 a AB	47.7 a AB	41.7 ab A	43.8 b A	41.0 b	41.5 b
	a*	3.5 B	3.7	3.9 C	4.4 A	4.0 A	3.7
	b*	19.3 a B	18.0 ab	16.8 ab AB	16.4 b B	16.3 b	16.0 b
	ΔE	0.9 d C	2.4 c D	4.6 b D	4.6 b C	5.5 a B	5.2 a C
	pH	6.0 B	6.0	5.8 C	5.7 A	5.6 A	5.7 A
US_50_10+ HHP_500_10	L*	48.5 a A	48.2 a A	42.3 b A	42.3 b B	40.5 b	41.4 b
	a*	4.4 A	4.2	4.2 AB	4.0 ABC	4.0 A	3.8
	b*	22.6 a A	21.1 a	18.1 ab A	17.5 ab A	16.7 b	16.7 b
	ΔE	4.7 B	3.5 BC	3.6 E	3.8 D	5.7 B	4.9 C
	pH	6.1 BC	5.9	6.0 BC	5.6 AB	5.7 A	5.6 A

All data shown are means (n=3); Standard deviations are shown in figures placed in the body of the manuscript. Indicators of the statistical analysis (Tukey test; p-value < 0.05): * small letters - significant differences in rows for the relevant parameters between storage times;* uppercase letters - significant differences in columns for the relevant parameters between treatments; the absence of letter in the same row or column indicates no significant differences between storage time or studied treatments (p-value > 0.05).

Table S2. Aerobic mesophilic bacteria, psychrophiles, mold & yeast, and enterobacteria (log CFU/mL) changes during 28 days at 4 °C in a broccoli and carrot by-products beverage according to several processing treaments.

Processing Treatment	Parameter	0 d	3 d	8 d	15 d	22 d	28 d
CTRL	Aerobic mesophilics	5.4 b A	4.9 b A	5.5 b A	5.9 b A	8.1 a A	8.5 a A
	Psicrophyles	4.5 c A	4.2 c A	5.0 c A	5.6 bc A	6.8 b B	8.5 a A
	Mold & Yeast	3.7 a A	3.7 a A	2.0 b	2.1 b	2.0 b	2.0 b
	Enterobacteria	3.8 a A	3.1 b A	2.7 bc	2.0 c	2.0 c	2.0 c
P_90	Aerobic mesophilics	4.0 a B	2.7 b B	2.8 b B	2.8 b B	2.3 bc B	2.0 c B
	Psicrophyles	3.4 a B	3.4 a C	2.8 ab B	2.0 b B	2.0 b C	2.1 b B
	Mold & Yeast	2.9 a B	2.7 a B	2.0 b	2.0 b	2.0 b	2.0 b
	Enterobacteria	2.7 a B	2.7 a B	2.7 a	2.0 b	2.0 b	2.0 b
HHP_500_10	Aerobic mesophilics	3.7 a B	2.7 b B	2.7 b B	2.0 a C	2.0 a B	2.0 a B
	Psicrophyles	2.8 a C	3.1 a D	2.7 a B	2.0 b B	2.1 b C	2.0 b B
	Mold & Yeast	2.8 a B	2.8 a B	2.0 b	2.0 b	2.0 b	2.0 b
	Enterobacteria	2.7 a B	2.7 a B	2.7 a	2.0 b	2.0 b	2.0 b
US_50_10	Aerobic mesophilics	5.1 c A	5.0 c A	5.0 c A	6.0 b A	7.9 a A	8.5 a A
	Psicrophyles	4.2 d A	3.8 d B	5.0 c A	5.7 c A	7.7 b A	8.7 a A
	Mold & Yeast	3.6 a A	3.5 a A	2.0 b	2.0 b	2.0 b	2.0 b
	Enterobacteria	3.6 a A	2.9 b AB	2.7 b	2.0 c	2.0 c	2.0 c
US_50_5+HHP_500_5	Aerobic mesophilics	3.5 a B	2.7 b B	2.7 b B	2.0 c C	2.0 c B	2.0 c B
	Psicrophyles	2.7 a C	2.9 a D	2.7 a B	2.0 b B	2.0 b C	2.0 b B
	Mold & Yeast	2.7 a B	2.7 a B	2.0 b	2.0 b	2.0 b	2.0 b
	Enterobacteria	2.7 a B	2.7 a B	2.7 b	2.0 b	2.0 b	2.0 b
US_50_10+HHP_500_10	Aerobic mesophilics	3.6 a B	2.7 b B	2.7 b B	2.0 c C	2.3 bc B	2.0 c B
	Psicrophyles	2.7 a C	2.7 a D	2.7 a B	2.0 b B	2.3 bc C	2.0 c B
	Mold & Yeast	2.7 a B	2.7 a B	2.0 b	2.1 b	2.0 b	2.0 b
	Enterobacteria	2.7 a B	2.8 a B	2.7 a	2.0 b	2.0 b	2.0 b

All data shown are means (n=3); Standard deviations are shown in figures placed in the body of the manuscript. Indicators of the statistical analysis (Tukey test; p-value < 0.05): * small letters - significant differences in rows for the relevant parameters between storage times;* uppercase letters - significant differences in columns for the relevant parameters between treatments; the absence of letter in the same row or column indicates no significant differences between storage time or studied treatments (p-value > 0.05).

Table S3. Free polyphenolic content (mg GAE/L), FRAP, and DPPH (mg TE/L) changes during 28 days at 4 °C in a broccoli and carrot by-products beverage according to several processing treaments.

Processing Treatment	Parameter	0 d	3 d	8 d	15 d	22 d	28 d
**CTRL**	FPC	247 a A	254 a A	227 a B	133 b C	117 bc C	105 c B
	FRAP	454 a A	424 a A	416 a A	190 b A	184 b A	193 b A
	DPPH	6022 a E	4944 b D	4440 c C	3514 d C	1697 e D	1390 f D
**P_90**	FPC	210 a C	200 ab D	150 b E	92 c D	65 d D	64 d C
	FRAP	320 a B	318 a B	163 b E	112 c C	85 d C	94 cd C
	DPPH	9781 a B	8531 b A	7474 c A	7064 d A	6073 e A	4171 f A
**HHP_500_10**	FPC	225 a B	220 a C	215 a C	156 b B	119 b BC	118 b AB
	FRAP	423 a A	413 a A	362 ab BC	171 b AB	143 b B	129 c BC
	DPPH	11443 a A	9090 b A	5742 c B	5696 c B	3714 d B	3151 d B
**US_50_10**	FPC	254 a A	234 a B	202 b D	134 c C	135 c B	132 c A
	FRAP	436 a A	350 ab AB	307 b C	181 c A	166 c AB	167 c AB
	DPPH	6071 a E	4803 b D	4524 b C	3421 c C	3288 c BC	1797 d CD
**US_50_5+HHP_500_5**	FPC	250 a A	246 a AB	251 a A	185 b A	169 b A	120 c AB
	FRAP	445 a A	356 ab AB	209 b D	158 c BC	143 c B	142 c B
	DPPH	6887 a D	5835 b C	3842 c D	2923 d D	2894 d C	2835 d BC
**US_50_10+HHP_500_10**	FPC	233 a B	232 a B	227 a B	159 b B	140 c AB	132 c A
	FRAP	443 a A	401 ab A	388 b AB	184 c A	172 c A	170 c AB
	DPPH	8731 a C	7187 b B	4265 c C	3724 d C	3097 d BC	2855 e BC

All data shown are means (n=3); Standard deviations are shown in figures placed in the body of the manuscript. Indicators of the statistical analysis (Tukey test; p-value < 0.05): * small letters - significant differences in rows for the relevant parameters between storage times;* uppercase letters - significant differences in columns for the relevant parameters between treatments.

Table S4. Range of individual identified carotenoids and glucosinolates content (mg/L) during 28 d refrigeration (from 0 to 28 d).

		CTRL		P_90		HPP_500_10		US_50_10		US_50_5 + HPP_500_5		US_50_10 + HPP_500_10	
		0 d	28 d	0 d	28 d	0 d	28 d	0 d	28 d	0 d	28 d	0 d	28 d
**Lutein**	**Carotenoids**	1.4	1.4	1.4	1.4	1.4	1.4	1.4	1.4	1.4	1.4	1.4	1.4
**13-cis-β-carotene**		1.5	1.6	1.5	1.4	1.5	1.5	1.5	1.5	1.5	1.6	1.6	1.5
**All-trans-β-carotene**		1.57	1.6	1.6	1.5	1.6	1.6	1.5	1.6	1.7	1.6	1.7	1.6
**9-cis-β-carotene**		1.4	1.4	1.4	1.3	1.4	1.4	1.4	1.4	1.4	1.4	1.4	1.4
**Glucoraphanin**	**Sulfur-compounds**	417	416	43.2	42.1	418	417	417	417	419	416	418	417
**4-Methoxy-glucobrassicin**		415	415	41.5	41.7	418	416	415	415	416	415	415	415
**Neoglucobrassicin**		416	422	41.4	42.0	416	423	417	424	417	428	416	425
**Sulforaphane**		5.2	5.5	4.0	4.3	5.5	5.7	5.23	5.9	5.3	5.8	5.4	5.7
